# Indoor–Outdoor Oxidative Potential of PM_2.5_ in Wintertime Fairbanks, Alaska: Impact of Air Infiltration and Indoor Activities

**DOI:** 10.1021/acsestair.3c00067

**Published:** 2024-02-14

**Authors:** Yuhan Yang, Michael A. Battaglia, Ellis S. Robinson, Peter F. DeCarlo, Kasey C. Edwards, Ting Fang, Sukriti Kapur, Manabu Shiraiwa, Meeta Cesler-Maloney, William R. Simpson, James R. Campbell, Athanasios Nenes, Jingqiu Mao, Rodney J. Weber

**Affiliations:** † School of Earth and Atmospheric Sciences, Georgia Institute of Technology, Atlanta, Georgia 30332, United States; ‡ Department of Environmental Health & Engineering, Johns Hopkins University, Baltimore, Maryland 21218, United States; § Department of Chemistry, University of California, Irvine, California, 92697, United States; ∥ Geophysical Institute and Department of Chemistry & Biochemistry, University of Alaska Fairbanks, Fairbanks, Alaska 99775, United States; ⊥ Laboratory of Atmospheric Processes and their Impacts (LAPI), School of Architecture, Civil & Environmental Engineering, Ecole Polytechnique Fédérale de Lausanne, Lausanne 1015, Switzerland; # Center for Studies of Air Quality and Climate Change, Institute of Chemical Engineering Sciences, Foundation for Research and Technology, Patras, Hellas 26504, Greece

**Keywords:** indoor air quality, subarctic region, residential heating, biomass burning, fine particulate matter (PM_2.5_), oxidative potential

## Abstract

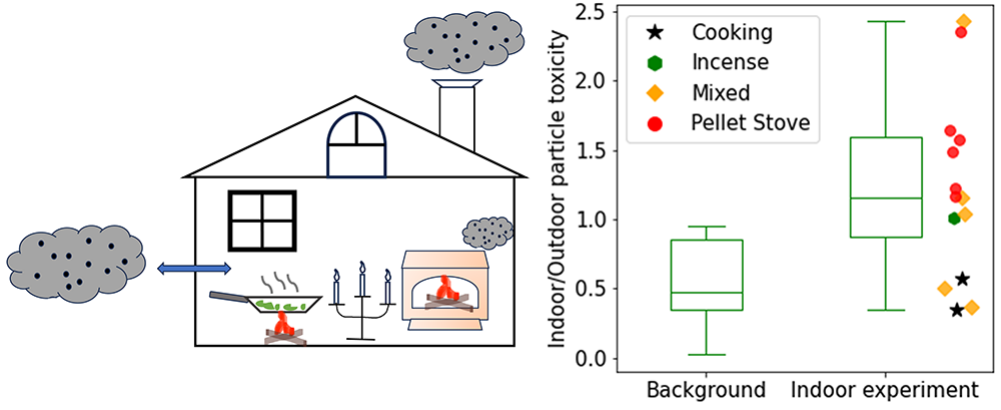

The indoor air quality of a residential home during winter in Fairbanks, Alaska, was investigated and contrasted with outdoor levels. Twenty-four-hour average indoor and outdoor filter samples were collected from January 17 to February 25, 2022, in a residential area with high outdoor PM_2.5_ concentrations. The oxidative potential of PM_2.5_ was determined using the dithiothreitol-depletion assay (OP^DTT^). For the unoccupied house, the background indoor-to-outdoor (I/O) ratio of mass-normalized OP (OP_m_^DTT^), a measure of the intrinsic health-relevant properties of the aerosol, was less than 1 (0.53 ± 0.37), implying a loss of aerosol toxicity as air was transported indoors. This may result from transport and volatility losses driven by the large gradients in temperature (average outdoor temperature of −19°C/average indoor temperature of 21 °C) or relative humidity (average outdoor RH of 78%/average indoor RH of 11%), or both. Various indoor activities, including pellet stove use, simple cooking experiments, incense burning, and mixtures of these activities, were conducted. The experiments produced PM_2.5_ with a highly variable OP_m_^DTT^. PM_2.5_ from cooking emissions had the lowest OP values, while pellet stove PM_2.5_ had the highest. Correlations between volume-normalized OP^DTT^ (OP_v_^DTT^), relevant to exposure, and indoor PM_2.5_ mass concentration during experiments were much lower compared to those in outdoor environments. This suggests that mass concentration alone can be a poor indicator of possible adverse effects of various indoor emissions. These findings highlight the importance of considering both the quantity of particles and sources (chemical composition), as health metrics for indoor air quality.

## Abstract 2

This study reveals that PM infiltrating from outdoor to indoor environments exhibited substantial changes in oxidative potential and that various indoor activities generated particles with a wide range of toxicity showing the importance of both the quantity and sources (chemical composition) of particles as health metrics for indoor air quality.

## 1 Introduction

The air quality in Fairbanks, a high latitude (64.84°N) city in Alaska’s interior, often exceeds fine particle (PM_2.5_) standards during the wintertime. This is mainly due to severe meteorological conditions that limit dispersion of emissions largely from residential heating with wood.^1−6^ With average outdoor low and high temperatures of nominally −24 °C and −15 °C in the wintertime, residents spend most time indoors. To some extent, this may isolate residents from the poor outdoor air quality but also intensify exposures to indoor emissions due to minimized indoor/outdoor air exchange.

Indoor air pollution has emerged as a significant global health concern, contributing to a substantial burden of disease worldwide. Household air pollution was responsible for an estimated 3.2 million deaths per year in 2020, including over 237 000 deaths of children under the age of 5.^7^ It can produce immediate health risks, such as headache and dizziness, and long-term health consequences, including respiratory infection, pulmonary diseases, cardiovascular disorders, and cancer.^8−11^ Indoor PM generated from different sources has been linked to premature death in people with heart or lung disease, nonfatal heart attacks, irregular heartbeat, aggravated asthma, decreased lung function, and increased adverse respiratory symptoms.^8^ Exposure to solid fuel smoke, for example, has been linked to chronic obstructive pulmonary disease in women, acute respiratory infection in children, lung cancer in women, and 3.5 million premature deaths.^12,13^ Obesity may increase susceptibility to the effects of indoor fine and coarse PM exposure.^14^ Across the globe, much of the exposure to indoor smoke is from low-efficiency traditional cooking methods with biomass-fuel, but in cold climates in many nations, indoor wood burning can be common as a source of heat or for recreation.

Indoor air quality is influenced by a combination of factors, including both outdoor air infiltration and emissions from indoor sources. Continuous exchange between outdoor and indoor air impacts indoor air quality, especially when outdoor air quality is poor, highlighting the significance of the outdoor air concentration and composition. This may contribute to the observed associations between outdoor air quality and health issues in epidemiological studies, even though people spend the most time indoors. During winter in Fairbanks, residential heating with fuel oil and wood has been identified as a significant contributor to outdoor PM_2.5_ mass concentration, followed by sulfate and vehicular emissions.^4^ The infiltration of ambient PM_2.5_ into indoor environments involves the movement of outdoor air into the house through openings, such as through gaps around windows and doors as well as through walls, floors, ceilings, and vents. Species that infiltrate are affected by concentration gradients between the indoor and outdoor environments, as well as differences in temperature and relative humidity (RH). Higher indoor temperature and lower indoor RH both can contribute to specific chemical species losing mass when moving from outdoors to indoors.^15,16^ In extremely cold regions like wintertime Fairbanks, where there are significant indoor-to-outdoor temperature and RH differences, this effect could be substantial.^17,18^

Indoor emissions of PM_2.5_ can be episodic and include PM resuspension,^19^ cooking,^20,21^ and combustion emissions, such as from stoves, fireplaces, or other forms of space heating or recreational burning.^22^ Secondary PM can also be generated from the reactions between volatile organic compounds (VOCs) and oxidants.^23^ VOCs can be emitted from indoor sources and from evaporation of infiltrating particles in a heated house during cold periods.^19,24,25^ Oxidants, such as ozone from outdoors and hydroxyl radical (OH·) and nitrate radical (NO_3_·) produced indoors,^25^ can react with VOCs, generating secondary species, but these are often a minor contributor to indoor PM_2.5_ mass concentration.^26^

Indoor emissions from burning wood are especially important in Fairbanks since wood is widely utilized for heating^3^ and can be a source of oxidative products.^27^ For example, pellet stoves can emit large amounts of fine particles^28^ and high levels of CO and polycyclic aromatic hydrocarbons (PAHs),^29^ especially in the ignition phase when combustion efficiency is low.^30,31^ Cooking is another major source of indoor aerosols, which can emit fatty acids and dicarboxylic acids,^32,33^ as well as combustion products if burning occurs.

These emissions can produce a substantial PM_2.5_ mass concentration, especially in a relatively small, tightly sealed house. Outdoor PM_2.5_ mass concentrations have been linked to adverse health effects. By also considering particle chemical composition, specific components of emissions, and any changes they may undergo through various processes that lead to more hazardous aerosols, can provide more insights than mass concentration alone.^34^ This may be especially important when considering indoor air quality since not all chemical components that substantially contribute to mass concentration are equally toxic. An alternative approach is to assess the ability of a particle to induce oxidative stress when inhaled. This results from the accumulation of reactive oxygen or nitrogen species (ROS/RNS) *in vivo* that cause cellular, tissue, and DNA damage,^35^ leading to a wide range of adverse effects.^36^ Various chemical assays have been developed to quantify an aerosol particle’s ability to generate ROS through the measurement of oxidative potential (OP). The dithiothreitol-depletion (DTT) assay is one of the most commonly used assays and has been linked to adverse effects, including both acute and chronic diseases.^37−41^ Other OP assays have also been associated with adverse health effects.^41−45^ OP^DTT^ is most sensitive to certain metals and organic species, such as Cu, Mn, and various aromatic species.^46−48^ It can be used to provide insight into potential dose (OP normalized by the volume of air, OP_v_) and the particle’s intrinsic health-relevant properties (OP normalized by particle mass concentration, OP_m_). A more comprehensive assessment of air quality is likely achieved by utilizing several complementary assays that are sensitive to different sources and aerosol species.^49−51^

Most studies on aerosol particle OP have focused on outdoor air, and we have reported on Fairbanks wintertime outdoor OP^DTT^.^51^ Fewer studies have explored OP of indoor particulate matter from indoor-outdoor air exchange^52,53^ and from various indoor sources.^54^ Here we report on the effects of infiltration of outdoor air and the intrinsic health-relevant properties of different indoor activities using the OP^DTT^ assay.

## 2 Methods

### 2.1 Sampling Location and House Description

This research was part of the Alaskan Layered Pollution And Chemical Analysis (ALPACA) field campaign. Indoor and outdoor daily PM_2.5_ samples were collected from 17 January 17, 2022 to 25 February 25, 2022, at the ALPACA House field site (64.850°N, 147.676°W) located in a Fairbanks residential area (Shannon Park Neighborhood). Residential areas of Fairbanks generally have higher concentrations of PM_2.5_ species emitted from residential heating, such as organic species from wood burning.^55^ The house was in a residential area and similar to other types of housing in Fairbanks.^56^ It was a single-level (ranch style) structure covering approximately 1549 square feet (excluding the garage). Prior to the intensive study, a house pressurization test and energy audit were conducted. The house was depressurized by 50 Pa, and leakage into the house was measured. A door blower test estimated an air exchange rate of 0.12/h under natural conditions. Additionally, the house ranked at the bottom of a 4-star rating (on a scale ranging from 1 to 6 stars), implying it was moderately above the typical Fairbanks residential housing standards in terms of air tightness, thermal resistance, and indoor ventilation patterns.

The main heating system was oil-burning with forced air distribution. The furnace blower fan was run continuously throughout the study to ensure adequate mixing of indoor air. The thermostat was set to 20 °C (68 °F). Online instruments (AMS and AE33) were sampled via small tubing from a hallway off the kitchen, whereas filters were collected in two rooms away from the kitchen and one room (through a doorway) from the pellet stove (see Figure S1), with the doorway being consistently open. Because this study reports nominally 24 h averaged data, we do not investigate the time evolution of indoor emissions or other perturbations.

### 2.2 PM_2.5_ Sampling

Separate systems were used for outdoor and indoor filter sampling. For the outdoor filters, a total of 49 PM_2.5_ filter samples (including 7 blanks) were collected using a Tisch PM_2.5_ high-volume (Hi-Vol) sampler (flow rate normally 1.13 m^3^/min). Each filter was collected over approximately 23.5 h (10 am to 9:30 am next day) using quartz filters (20.32 × 25.40 cm; Whatman® QM-A quartz filter). Inside the house, 35 PM_2.5_ filter samples were collected during the same period and synchronized with the outdoor sampling times using a PM_2.5_ Partisol-Plus sampler (Model 2025 sequential air sampler, Rupprecht & Patashnick Co., Inc, flow rate normally 16.7 L/min) with Teflon filters (46.2 mm in diameter with PP ring supported, pore size of 2 μm, Whatman® PTFE membrane filter). Neither set of filters was denuded of gases. A previous study found no statistically significant difference when comparing PM_2.5_ OP^DTT^ collected on quartz filters to that collected on Teflon filters,^57^ although another study reported a 21% reduction in OP_v_^DTT^ associated with quartz filters relative to Teflon filters.^58^ The collected samples were stored at −20 °C until analysis, which occurred approximately 100 days following the study.

### 2.3 Acellular Oxidative Potential Measurements

Both indoor and outdoor filter samples were analyzed for total OP by the DTT depletion assay (OP^DTT^) for PM_2.5_ particles. (This is often referred to as OP^total DTT^. The OP measurement method used in this study was designed to include both insoluble and soluble particle components, in contrast to the often-measured water-soluble fraction.) A fraction from each filter was placed in a sterile polypropylene centrifuge vial (VWR International LLC, Suwanee, GA, USA) for extraction and analysis. Due to the possible nonlinear response of the DTT assay to extract mass,^46^ the punched filter fraction and the volume of water used for extraction were adjusted, based on the PM_2.5_ mass loading on each filter, to achieve a relatively constant extract particle concentration of 10 μg/mL for both indoor and outdoor filters. Extraction of filters was performed using deionized Milli-Q water (DI, Nanopure InfinityTM ultrapure water system; resistivity > 18 MΩ/cm) via sonication (Ultrasonic Cleanser, VWR International LLC, West Chester, PA, USA) for 60 min. The filter punch remained in the extracts throughout the OP analysis to allow insoluble species to interact with the reagents. Details of the established protocol can be found in Gao et al. (2017).^57^ Both volume-normalized (OP_v_^DTT^) and mass-normalized (OP_m_^DTT^) results are discussed. Details of the outdoor OP^DTT^ can be found in Yang et al. (2024).^51^

### 2.4 Aerosol Mass and Composition Measurements

The online instrumentation was in an attached garage but isolated from the house (Figure S1). They were equipped with continuous flow switching inlets to consecutively sample indoor/outdoor air. PM_1_ composition measurements were made with a High-Resolution Time-of-Flight Aerosol Mass Spectrometer (HR-ToF-AMS, Aerodyne Research, Inc., USA)^15^ with a 10 min indoor/10 min outdoor sampling cycle. Following a Nafion dryer, the HR-ToF-AMS measured non-refractory PM_1_ species, including NH_4_^+^, NO_3_^–^, SO_4_^2–^, Cl^–^, and organic aerosol (OA). Indoor and outdoor light-absorbing particles were measured by an aethalometer (AE-33, Magee Scientific, Berkeley, CA) at 1 Hz by employing the same I/O switching inlet. To align with the start/stop times of the AMS, the AE-33 time series data were converted to 1 min averages for subsequent analysis. These datasets were then merged to the filter sampling times and separated into indoor and outdoor sampling periods. Light absorption measured at the 880 nm wavelength was used to determine the mass equivalent black carbon (BC) concentration based on the manufacturer’s stated mass absorption cross-section.

Neither indoor nor outdoor PM_2.5_ mass concentration was directly measured. Indoor PM_2.5_ mass concentration was determined by summing the AMS-measured non-refractory PM_1_ species and AE33-measured BC and merging them to the filter sampling time (24 h). This estimate was in reasonable agreement with the PM measurements using a medium-cost MODULAIR-PM sensor which combines a nephelometer and optical particle counter to measure PM_1_, PM_2.5_, and PM_10_ after correcting for humidity^59^ (Figure S2, slope 0.91, intercept 1.36 μg/m^3^, *r*^2^ = 0.92). For outdoor PM_2.5_ mass estimation, the AMS-measured species were combined with elemental carbon and metals analyzed from the filters, as described in Yang et al. (2024).^51^ This estimation showed good agreement with the PM_2.5_ mass concentrations measured by the Alaska Department of Environmental Conservation for the U.S. Environmental Protection Agency using a Beta Attenuation Monitor (BAM) at the National Core (NCore) monitoring site (roughly 2.6 km from the house) (slope 1.04, intercept 2.07 μg/m^3^, *r*^2^ = 0.70). While a similar assessment of the indoor PM_2.5_ mass concentration was not possible, missing species in the indoor measurement, such as metals and other refractory species, contributed only a small fraction (about 1%) to outdoor PM_2.5_ mass concentration. Therefore, these components were also expected to have a limited impact on the mass of indoor aerosol particles.

### 2.5 Indoor Experiments

The study involved various indoor activities, such as heating with a pellet stove, simple stovetop (electrical heating) cooking activities, and burning incense. The pellet stove was an open-hearth fireplace insert and was installed just prior to the study. It experienced issues throughout the study with exhaust leakage at the point where the pellet stove exhaust pipe connected to the existing chimney flue. This resulted in indoor smoke levels that were higher than is typically expected for this stove. The simple cooking experiments mainly involved the generation of aerosols from fats and oils from stove-top heating of vegetable oil with or without added food (e.g., chicken), as well as heating pasta and noodles. Specific details are given in Table S1. The particles and gases produced by these various indoor activities could interact with both the infiltrating air and other indoor components and surfaces. Mixed experiments, involving simultaneous cooking and pellet stove activities, with both contributing to indoor PM emissions, were also conducted (see Table S1). The house was unoccupied when there were no experiments or necessary instrument-related activities. This allowed for a focused analysis during periods when infiltration was the primary source of indoor particulate matter.

## 3 Results

### 3.1 Indoor PM Oxidative Potential

Throughout the study period, for a house with minimal indoor activities, the average indoor volume-normalized oxidative potential (OP_v_^DTT^) was 0.12 ± 0.10 nmol/min/m^3^ (mean ± standard deviation). This amount was approximately one-fourth of the corresponding average of the outdoor value (0.418 ± 0.215 nmol/min/m^3^), indicating significantly reduced exposures to OP of PM indoors. However, the mean intrinsic OP values (toxicity) of particles during the entire study period were comparable for indoor and outdoor settings, with OP_m,in_^DTT^ = 31 ± 19 pmol/min/μg and OP_m,out_^DTT^ = 35 ± 17 pmol/min/μg, meaning that, on average, the toxicity (measured by this assay) of the aerosol was similar between indoors and outdoors.

### 3.2 Effects of Outdoor Air Infiltration on OP^DTT^: Insights from Comparison with Sulfate

To determine the effect of infiltration of outdoor air on indoor particle health-related properties, we compared the indoor-to-outdoor (I/O) ratio of PM_1_ sulfate and PM_2.5_ OP^DTT^ based on 24 h average data. Assuming sulfate was nonvolatile, lost only by mass-transport processes (e.g., impaction, interception, diffusion), and had no indoor sources, differences in I/O ratios of other PM species compared to sulfate would indicate changes due to gas/particle partitioning, or contributions from indoor sources, or both.^15^

Comparisons are made for two periods, both when there was no activity in the house: (1) Background periods, the time at the beginning of the study before any perturbation experiments; (2) No-experiment periods, the time between perturbation experiments throughout the study. See Figure S3 for a time series of the study, with periods of indoor perturbation experiments identified.

The average I/O ratio of sulfate during the combined Background and No-experiment periods was 18% (Figure S4, regression results of I/O ratio of sulfate; slope = 0.183, intercept = 0.049, *r*^2^ = 0.91), demonstrating that during the infiltration of outdoor air into the house, most of the sulfate-contained particles were removed, effectively lowering indoor exposures by about 80%. Similarly, BC is nonvolatile and may be externally mixed with some fraction of the sulfate, meaning it would potentially have a similar I/O ratio.^15,16^ Unfortunately, even after an inline nafion drier upstream of the aerosol instruments, the T and RH variation between indoor and outdoor sampling significantly reduced the accuracy of determining BC from the AE-33 measurement, making the calculations of the I/O ratio of BC less accurate. Hence, we assumed a similar I/O ratio as sulfate for BC and other nonvolatile PM components. Particle losses due to deposition during transport are size-dependent,^60^ potentially leading to varying removal efficiencies for different PM components compared to sulfate during the infiltration process, which has been observed in previous indoor/outdoor studies.^15,16^ This effect is likely small since by mass most particle chemical components of PM_2.5_ (e.g., sulfate, water-soluble metals, and organic aerosol) were in the size range of 0.1 to 1 μm. For semivolatile aerosol species, substantially lower I/O ratios would be expected due to the evaporation of these semi-volatile components, driven by the large I/O temperature and RH gradients. For the study period, the outdoor temperature ranged from roughly −35 °C to 5 °C, and outdoor RH ranged from 47% to 97% (1-h average). The indoor temperature ranged from 19.4 °C to 24.4 °C, and indoor RH ranged from 8.6% to 15.6% (1-h average).

**Figure 1:**
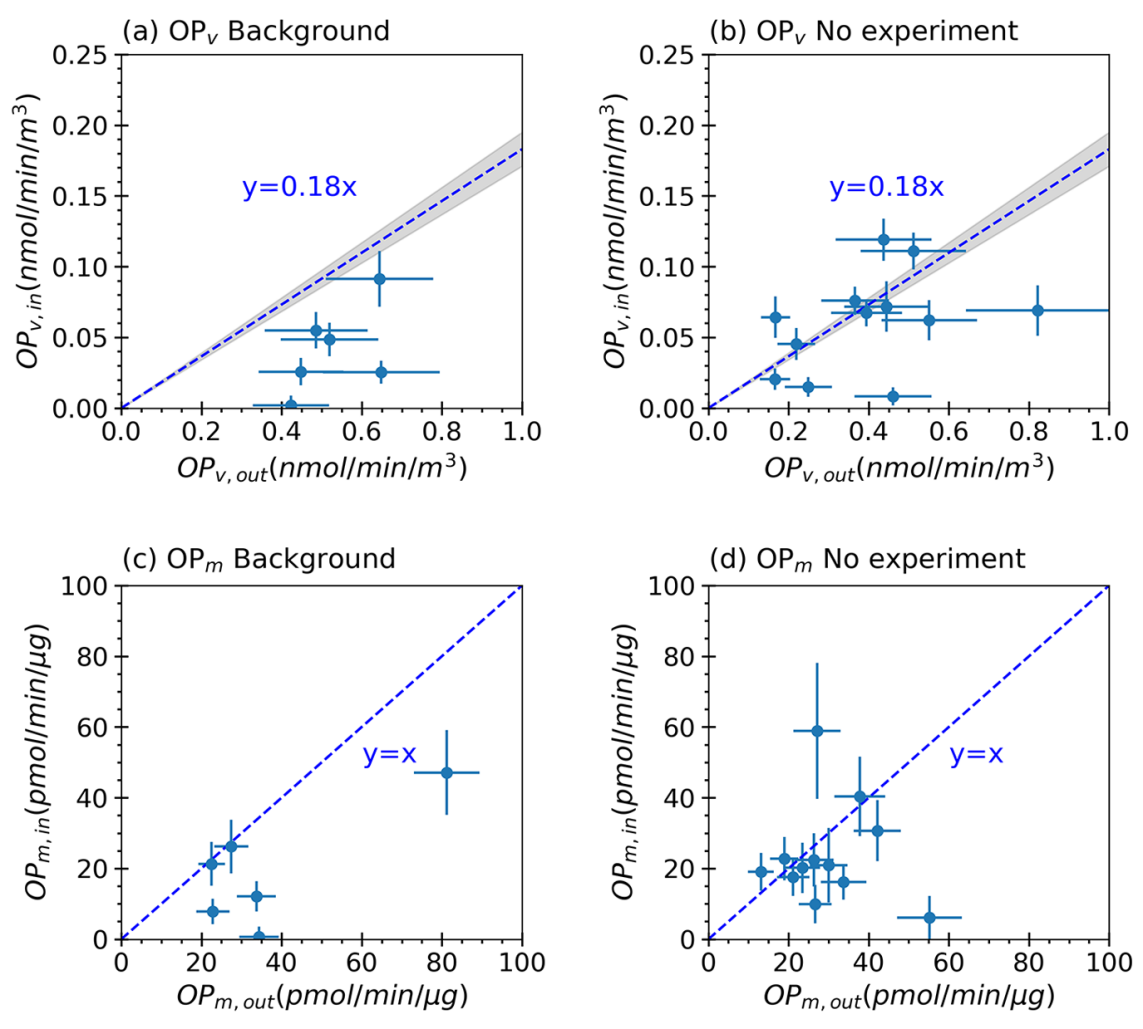
Indoor versus outdoor levels of volume-normalized and mass-normalized OP determined with the DTT assay for both Background and No-experiment periods. Panels (a) and (b) depict volume-normalized OP, while panels (c) and (d) show mass-normalized OP. The shaded gray regions within (a) and (b) correspond to areas calculated based on the uncertainty arising from the regression slope of indoor versus outdoor PM_1_ sulfate concentrations (as shown in Figure S3). The error bars denote the measurement uncertainty for OP.

OP_v_^DTT^ appeared to be influenced by the loss of semivolatile species that the DTT assay is sensitive to. For the Background period (prior to any indoor activities), the indoor OP_v_^DTT^ relative to outdoor OP_v_^DTT^ was consistently below the 18% observed for sulfate (Figure 1a). This was also observed when comparing the I/O ratio of mass-normalized OP^DTT^ (OP_m_^DTT^), shown in Figure 1c, where data points were on or below the 1-to-1 line.

We have shown that outdoor OP^DTT^ is sensitive to BC, biomass-burning organic aerosol (AMS-determined BBOA) species, and copper (Cu).^51^ It is likely that specific species associated with BBOA, such as oxygenated PAHs originating from biomass burning emissions,^61−63^ including quinones—to which the DTT assay is known to be very responsive^64^—may exhibit varying levels of volatility. Their particle-phase concentrations could be negatively correlated with temperature.^65−67^

However, no correlation was observed between the I/O ratio of OP_v_^DTT^ and outdoor BBOA (*r* = 0) or outdoor temperature (*r* = 0.09). This lack of correlation is likely due to additional factors, such as variations in particle composition and changes in indoor/outdoor relative humidity (RH).

The infiltration process led to aerosol drying that would result in a reduction in water content due to the significant I/O RH gradient (mean of ΔRH = −65%). This could cause the evaporation of certain water-soluble DTT-responsive species, such as benzoquinone, since the outdoor OP_v_^DTT^ was largely water-soluble (mean water-soluble to total was 77%).^51^ The I/O ratio of OP_v_^DTT^ was negatively correlated with outdoor RH (*r* = −0.77); a larger I/O ratio of OP_v_^DTT^ was observed when outdoor RH was lower and closer to the relatively constant indoor RH of about 10%. The I/O ratio of OP_v_^DTT^ was positively correlated with outdoor EC and Cu (*r* = 0.78 for both), both of which are nonvolatile and expected to behave similarly to sulfate during air infiltration. These findings suggest that a significant decrease in RH and variability in particle composition affected the volatility losses, consequently impacting indoor OP levels. We used concentration of outdoor PM_2.5_ components here for the correlation analysis since some indoor chemical species, including EC and metals, were not measured due to insufficient PM mass collected on our filters to enable their analysis. A difference in the transmission efficiency of the gas-phase PAHs and related species relative to corresponding PM_2.5_ species may also alter the equilibrium when air moves between indoors and outdoors, but this is likely a minor effect relative to the extreme I/O differences in temperature and RH observed in this study.

The overall effect is that the health-related properties of the infiltrated outdoor PM_2.5_, as measured by the DTT assay, were substantially lower. This is despite the use of a filter sampling system to collect the particles for the OP measurement, which is in general known to miss more short-lived species, such as semivolatile organic compounds^68,69^ that may contribute to OP^DTT^,^70^ and that the filters had been archived for an extended period prior to analysis. It is likely that the I/O Δ*T* and ΔRH values were so high in this case that the effect was observed for a filter sampling system.

A similar analysis was performed for the No-experiment periods (Figure 1b,d). Indoor experiments were performed during the daytime, and measurements of different VOCs showed that VOC concentrations decay exponentially within 1 to 4 h after emissions ceased (e.g., when cooking ended or the pellet stove was shut off, etc.). There were 12 periods when the 24-h filter samples were not directly affected by the perturbation experiments from the prior days (see Figure S3). For these No-Experiment periods, most of the I/O ratio of OP_v_^DTT^ data points were either close to or below the sulfate ratio line (Figure 1b), and for OP_m_^DTT^, they clustered near or below the unity slope line (Figure 1d). This pattern resembled the Background results, but there was greater dispersion in the No-experiments data. This dispersion could be due to more data points, or the residual presence of pollutants emitted in previous-day experiments. These pollutants may be adsorbed by indoor surfaces, subsequently repartitioning to particles, or lead to the production of secondary species that repartitioned to the particles, both cases raising the indoor OP^DTT^. The long sample time of these OP^DTT^ data limits a more detailed investigation of the repartitioning of the DTT-active species. We speculate this partitioning would be mainly from pellet stove emissions adsorbing to indoor surfaces, providing a reservoir for subsequent re-emission.^71^ From these data, we cannot conclude that there is a definitive effect. In the further analysis below, given the substantial variability in OP^DTT^ observed during the No-experiment period, the pollutant enhancements and PM_2.5_ OP^DTT^ in perturbation experiments were exclusively compared to the Background levels.

### 3.3 PM_2.5_ Mass Concentration and OP^DTT^ for Specific Indoor Perturbation Experiments

The perturbation experiments do not necessarily represent indoor concentrations for a typical household performing these various activities. Therefore, we compared the intrinsic health-relevant properties of these activities by focusing on OP_m_^DTT^ and compared correlations between OP_v_^DTT^ and PM_2.5_ mass concentrations. However, indoor OA concentrations can influence partitioning, potentially altering the relative concentration of components contributing to OP and, consequently, mass-normalized OP, which is not considered in this study.

**Figure 2:**
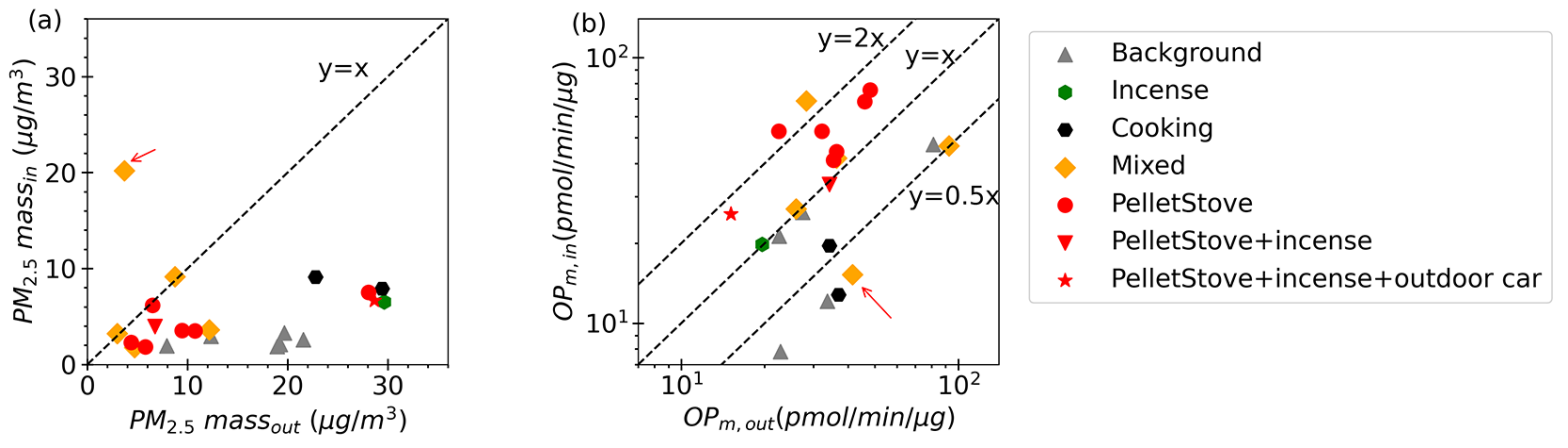
Comparison of (a) PM_2.5_ mass concentration and (b) OP_m_ (log scale) between indoor and outdoor environment based on 24-h averaged data. Note that one background data point is not plotted in (b) since lower than the range of the vertical axis plotted. The outlier of high PM_2.5_ indoor mass highlighted with an arrow in (a) is also identified in plot (b).

Some indoor perturbation experiments produced notably elevated PM_2.5_ mass concentrations (1 min average, as per AMS + BC data, peaked at 754 μg/m^3^ during a mixed experiment.). These peaks were of limited temporal extent, so when averaged over the 24 h filter sampling time, the concentrations were much lower. Thus, the majority of indoor PM mass had concentrations of less than 10 μg/m^3^, with values either less than or equal to the 24-h average outdoor PM mass (Figure 2a).

When compared to Background conditions within the house, the mean PM_2.5_ mass concentration was higher for all perturbation experiments (Table 1). Cooking and mixed experiments had the most pronounced elevation; the mean PM_2.5_ mass concentration exceeded the Background level by more than a factor of 3. Pellet stove experiments produced a smaller increase relative to that in the Background (Table 1). The highest 24 h average indoor PM mass concentration of 20.18 μg/m^3^ occurred during a mixed experiment when both cooking activities and pellet stove emissions contributed to the indoor PM levels (the outlier in Figure 2a identified with an arrow, where very high cooking emissions occurred when water dripped into a pan of hot cooking oil, but no cooking activities took place).

**Table 1:** Indoor PM_2.5_ Mass Concentration, Volume- and Mass-Normalized OP^DTT^ and Indoor-to-Outdoor (I/O) Ratio of OP_m_^DTT^ during Background and Indoor Perturbation Experiments Based on 24-h Average Filter Samples^a^

	*N*	PM_2.5_ mass (μg/m^3^)	OP_v_^DTT^ (nmol/min/m^3^)	OP_m_^DTT^ (pmol/min/μg)	I/O OP_m_^DTT^
Background	6	2.45 ± 0.58	0.041 ± 0.031	19.2 ± 16.5	0.53 ± 0.37
No-experiment	12	2.86 ± 1.76	0.061 ± 0.034	23.8 ± 14.2	0.90 ± 0.54
Pellet Stove	6	4.14 ± 2.23	0.232 ± 0.140	55.9 ± 13.5	1.57 ± 0.43
Pellet Stove + incense	1	3.96	0.132	33.4	0.98
Pellet Stove + incense + outdoor car	1	6.67	0.172	25.8	1.71
Incense	1	6.53	0.130	19.8	1.01
Cooking	2	8.50 ± 0.85	0.136 ± 0.027	16.2 ± 4.8	0.46 ± 0.16
Mixed	5	7.58 ± 7.59	0.195 ± 0.079	39.9 ± 20.3	1.10 ± 0.82

a Means and standard deviations are shown.

Like PM_2.5_ mass, there was a noticeable increase in indoor OP related to exposure (OP_v_^DTT^) throughout all of the indoor perturbation experiments compared to the Background level (Table 1), highlighting the substantial possible health impact of indoor activities. Notably, experiments involving the use of the pellet stove had the most significant increase in OP_v_^DTT^ compared to that of the Background level, reaching an average of 0.232 nmol/min/m^3^. Conversely, particles generated from cooking and incense had comparatively lower levels of OP_v_^DTT^, despite cooking producing the highest PM_2.5_ mass concentration. Comparing differences in PM_2.5_ mass concentration between the various experiments to the Background levels and corresponding OP_v_^DTT^, shows no trend between PM_2.5_ mass concentration and OP_v_^DTT^ (Table 1), pointing to highly variable OP_m_^DTT^ between the different indoor emissions tested.

Another way to assess the relative OP^DTT^ of the indoor experiments is to compare the OP_m_^DTT^ (i.e., OP_v_^DTT^ divided by the indoor PM_2.5_ mass concentration) for each indoor experiment to the outdoor OP_m_^DTT^. This comparison shows the intrinsic OP properties (like toxicity) of indoor air relative to that of outdoor air. Figure 2b shows large differences between indoor and outdoor OP_m_^DTT^ values among perturbation experiments. Pellet stove-based experiments produced higher average OP_m_^DTT^ relative to outdoor levels (data points above the 1:1 line in Figure 2b and I/O ratio of OP_m_^DTT^ > 1 in Table 1). All cooking experiments resulted in OP_m_^DTT^ lower than outdoor levels (I/O ratio of OP_m_^DTT^ < 1). The OP_m_^DTT^ levels in cooking emitted particles were comparable with a previous study on both primary and secondary emissions from heated cooking oils (5–20 pmol/min/μg).^72^ In addition, the low toxicity of cooking-emitted particles was consistent with OP measurements obtained through electron paramagnetic resonance (OP^EPR^) in another study.^73^ Toxicity of incense-burning particle emissions closely resembled that of outdoor air (I/O ratio of OP_m_^DTT^ ≈ 1). OP_m_^DTT^ of the incense-burning particles was notably lower than that in a previous study (65.3–68.3 pmol/min/μg),^54^ likely attributed to the extended sampling time (24-h) in this study, averaging the toxicity of incense burning over a longer period relative to the 50 min burning time. Mixed experiments were scattered between these ranges, dependent on the relative amounts of pellet stove and cooking emissions. For example, the mixed experiment with extremely high PM_2.5_ mass concentration due to large cooking oil emissions (data point noted by the arrow in Figure 2a) had a low OP_m_^DTT^ (Figure 2b).

**Figure 3:**
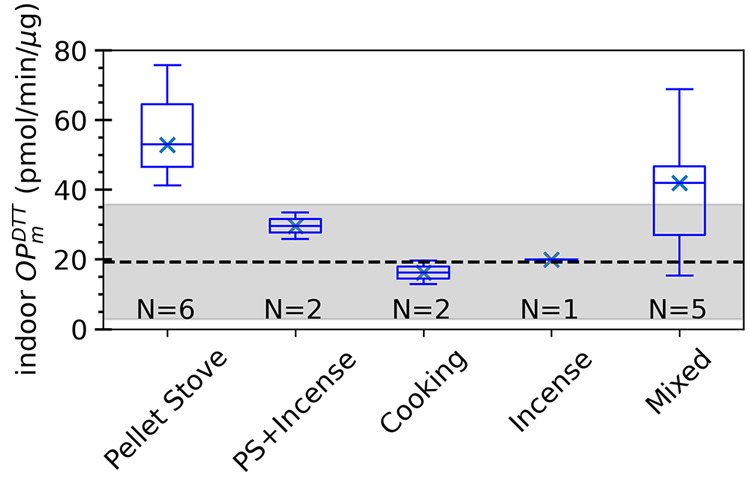
Boxplots of indoor OP_m_^DTT^ during different perturbation experiments relative to indoor Background OP_m_^DTT^, where Background is from measurements in the empty house prior to any indoor experiments and only due to infiltration of outdoor air. The mean Background OP_m_^DTT^ is indicated by the dashed line, and the shaded region represents its standard deviation. The two categories, pellet stove + incense and pellet stove + incense + outdoor car, with only one data point each, have comparable OP_m_^DTT^ levels and have been merged (PS + Incense) in this plot. The marker and line in the box indicate the median value (Q2), the lower and upper box boundaries indicate the first quartile (Q1) and the third quartile (Q3), and the whiskers indicate the minimum and maximum values for each corresponding category.

Differences in PM_2.5_ OP_m_^DTT^ (toxicities) for the various indoor activities and relative to the background levels due to outdoor air infiltration are shown in Figure 3. The pellet stove emissions had the highest OP_m_^DTT^ (average of 55.9 pmol/min/μg, Table 1), followed by mixed experiments involving pellet stove plus cooking and pellet stove plus incense emissions. Cooking emissions were associated with the lowest toxicity (mean OP_m_^DTT^ of 16.2 pmol/min/μg), even below the Background OP_m_^DTT^ levels when only infiltration of outdoor air contributed to indoor OP^DTT^. This is because the OP_v_^DTT^ was low for cooking, although the mass concentration was high. These intrinsic OP^DTT^ values were consistent with those reported by Bates et al. (2019), summarizing results from multiple studies.^74^ The OP_m_^DTT^ associated with pellet stove emissions fell within the range of values associated with biomass burning (20–200 pmol/min/μg), while cooking emissions were comparable with the lower limit of biogenic secondary OA (1–50 pmol/min/μg). Mixed experiments were similar to the average outdoor OP_m_^DTT^ of PM_2.5_ reported in a number of studies (nominally 30 pmol/min/ug).^74^

Overall, these results highlight the significance of considering both the chemical composition and the quantity (mass concentration) of particles as health metrics for indoor air quality.

### 3.4 Relationship between PM_2.5_ Mass Concentration and OP^DTT^

**Figure 4:**
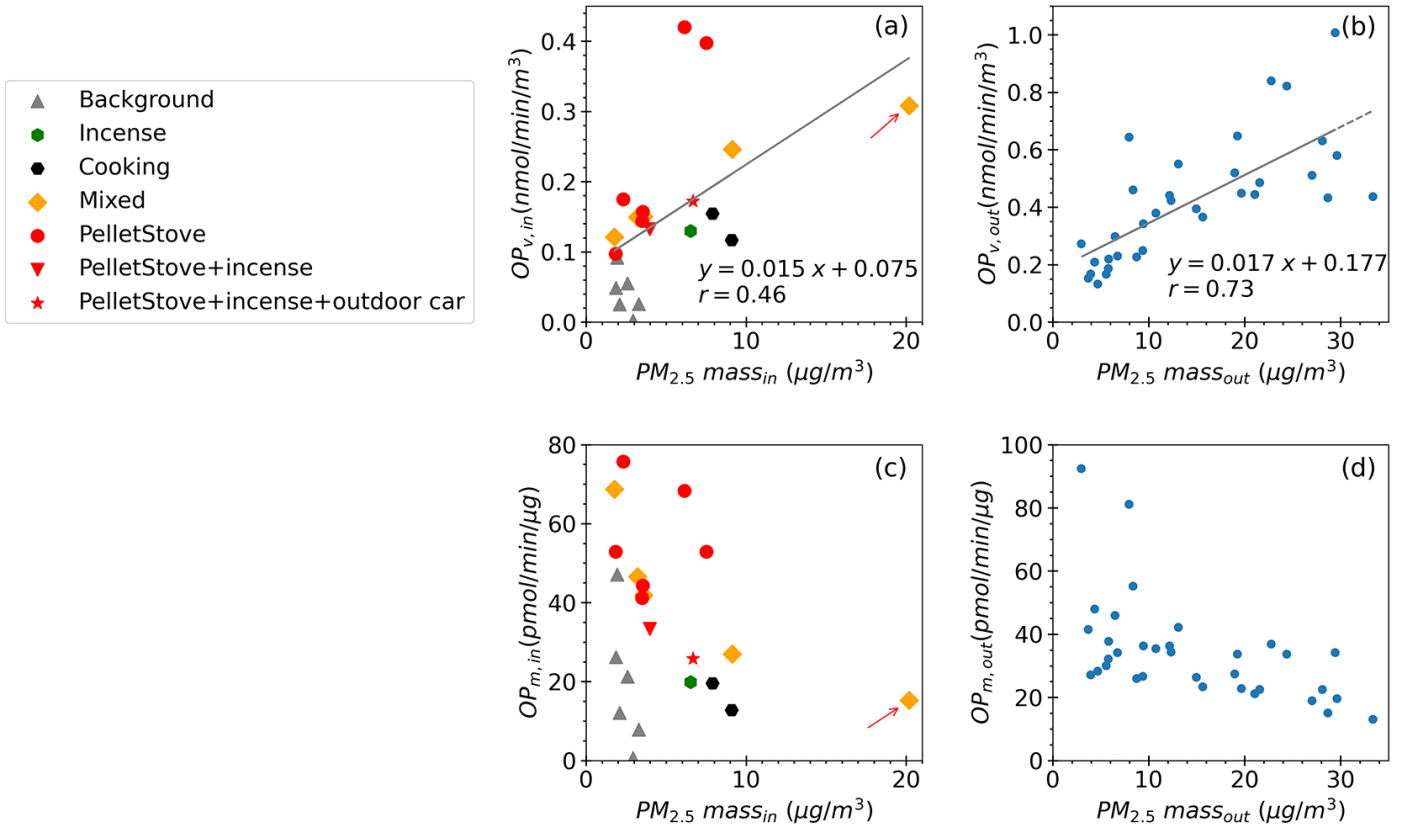
Scatter plots between indoor PM_2.5_ mass concentration and (a) indoor OP_v_ (c) indoor OP_m_ during different perturbation experiments, and between outdoor PM_2.5_ mass concentration and (b) outdoor OP_v_, (d) outdoor OP_m_ during the whole study period based on 24-h averaged data. Orthogonal regression was applied in (a) and (b). The outlier of high PM_2.5_ indoor mass highlighted with an arrow in (a) is also identified in plot (c).

Given that effects of PM on health are often based on correlations between PM_2.5_ mass concentrations and adverse health end points, correlations between PM_2.5_ mass concentration and OP_v_ are of interest. Comparing Figure 4a,b shows a much lower Pearson correlation between the OP_v_^DTT^ and PM_2.5_ mass concentration for indoor experiments (*r* = 0.46) compared to outdoor air (*r* = 0.73). The scattering of indoor data is attributed to the differences in various types of indoor emissions. The two data points significantly deviating from the regression line with relatively high OP_v_^DTT^ values in Figure 4a correspond to emissions from the pellet stove, indicating heightened toxicity. Moderate to high correlations (e.g., often in the range of 0.44 to 0.75) between OP_v_^DTT^ and PM mass concentration have been reported in ambient (outdoor air) studies.^48,75,76^ These findings, along with the considerable variability in OP_m_^DTT^ for the indoor experiments, suggest that the PM_2.5_ mass concentration may not adequately represent the relative adverse health responses from exposures to different indoor sources.

Correlations between OP_m_ and PM_2.5_ mass concentration can also show the extent of all PM_2.5_ species on particle toxicity. Both indoor and outdoor OP_m_ were negatively correlated with PM_2.5_ mass (Figure 4c,d), indicating that many PM components that contributed to PM_2.5_ mass did not significantly contribute to the OP response. Conversely, there were other PM components that contributed to the OP but had minor contributions to the overall PM_2.5_ mass concentration. Specifically, in terms of sources, pellet stove emissions were mainly concentrated in the upper left of Figure 4c, indicating relatively low PM loadings and high intrinsic toxicity. In contrast, cooking emissions in the middle to lower part of Figure 4c produced large amounts of PM (about 10 μg/m^3^) with an OP_m_ less than 20 pmol/min/μg. The mixed experiment involving concurrent emissions from both pellet stoves and cooking, showed the properties of both sources. The mixed experiment with very high cooking emissions (aerosolized hot oil in a pan, the outlier identified by the arrow in Figure 4) had a low OP_m_^DTT^.

## 4 Limitations and Implications

The indoor perturbation experiments of this study do not necessarily reflect a “typical” indoor home environment. All indoor experiments were conducted mainly in the afternoon and had relatively short durations. The cooking activities were highly simplified, and there were no other concurrent human activities. The pellet stove was not operated for extended periods and often produced high levels of smoke indoors despite being recently installed and serviced during the study. Pellet stove indoor emissions are expected to be much lower than other indoor wood-heating methods since the combustion chamber is isolated from the room, in contrast to open-hearth fireplaces and airtight stoves that require opening for loading wood (although ideally, a properly functioning airtight stove, once warmed, draws room air up the hot chimney, reducing emissions into the room during fueling).

The DTT assay utilized in this study, while sensitive to a wide range of chemical components, primarily focuses on the formation of superoxide (O_2_^•–^) and does not include the generation of hydroxyl radicals (·OH), which are crucial steps in the ROS cascade.^77^ Furthermore, DTT is a surrogate for reducing agents in cells, (e.g., NADH and NADPH)^78^ and may not perfectly replicate the biological processes of oxidative stress *in vivo*. Future research should incorporate multiple assays, such as assays capable of measuring PM’s ability to generate ·OH, or cellular OP assays that consider additional biological responses involving the generation of ROS within biological organisms, to better assess the health-relevant properties of indoor PM.

Although OP_v_^DTT^ values reported here may not be broadly comparable to other houses or locations, the Background and No-experiment cases may be representative of what occurs in other houses in Fairbanks and in other cold urban environments. OP_m_^DTT^ for the perturbation experiments provides a measure of the intrinsic health-relevant properties of these emissions (based on this assay) and can be compared to values reported in the literature for various other types of sources.^79^ The OP_m_^DTT^ (toxicity) of the higher-than-normal emitting pellet stove may be similar to emissions from other forms of wood-burning, which are expected to be high.^80^ Based on the DTT assay, we find that incomplete combustion emissions are of greatest concern, for both outdoor and indoor environments, which is well-established for outdoor pollution.

These OP data also enable comparisons to the PM_2.5_ mass concentration as an air quality metric. Several studies for outdoor aerosols (ambient air) have shown that OP may be more strongly linked to specific health end points than PM_2.5_ mass concentration.^49,79^ The indoor experiments show that divergence between OP and mass may be even greater for emissions in an indoor environment due to strong influences from individual sources, many of which may be non-combustion related and can have lower OP_m_^DTT^. More studies on the I/O ratio of OP in various locations using multiple assays are needed. Online measurement approaches would allow the assessment of volatile OP species^81−83^ and provide larger datasets. Overall, this study demonstrated the complex interplay between indoor and outdoor aerosol sources on indoor air quality characterized by the PM_2.5_ oxidative potential.

## Data Availability

Data is available on arcticdata.io: https://arcticdata.io/catalog/view/doi%3A10.18739%2FA23R0PV7J.

## Supporting Information Available

Detailed description of the indoor experiments (Table S1); 3-D rendering of the one-story house’s plan (Figure S1); comparison between indoor PM_2.5_ mass concentration estimated by PM_1_ + BC and PM_2.5_ measurement using a low-cost optical PM sensor (Figure S2); time series of indoor–outdoor (I/O) PM_2.5_ mass, I/O OP_m_^DTT^, and the time slot of different experiments (Figure S3); daily average PM_1_ sulfate concentrations measured indoors and outdoors with an Aerosol Mass Spectrometer merged to the filter sampling time during periods of no indoor activities (Figure S4) (PDF)
