# Supplementary material for: Indoor–Outdoor Oxidative Potential of PM2.5 in Wintertime Fairbanks, Alaska: Impact of Air Infiltration and Indoor Activities
Source: ACS EST Air. 2024 Feb 14;1(3):188–99. doi: 10.1021/acsestair.3c00067 (PMC10928657; doi:10.1021/acsestair.3c00067)
Supplement: Supplementary file 1 — ea3c00067_si_001.pdf [file ea3c00067_si_001.pdf]

Indoor - Outdoor Oxidative Potential of $PM_{2.5}$ in Wintertime Fairbanks, Alaska: Impact of Air Infiltration and Indoor Activities

Yuhan Yang[1], *Michael A. Battaglia*[1, a], *Ellis S. Robinson*[2], *Peter F. DeCarlo*[2], *Kasey C. Edwards*[3], *Ting Fang*[3, b], *Sukriti Kapur*[3], *Manabu Shiraiwa*[3], *Meeta Cesler-Maloney*[4], *William R. Simpson*[4], *James R. Campbell*[4], *Athanasios Nenes*[1, 5, 6], *Jingqiu Mao*[4], *Rodney J. Weber*[1, *]

[1] School of Earth and Atmospheric Sciences, Georgia Institute of Technology, Atlanta, Georgia 30332, USA

[2] Department of Environmental Health & Engineering, Johns Hopkins University, Baltimore, Maryland, 21218 USA

[3] Department of Chemistry, University of California, Irvine, California, 92697 USA.

[4] Geophysical Institute and Department of Chemistry & Biochemistry, University of Alaska Fairbanks, Fairbanks, Alaska, 99775 USA

[5] Laboratory of Atmospheric Processes and their Impacts (LAPI), School of Architecture, Civil & Environmental Engineering, Ecole Polytechnique Fédérale de Lausanne, Lausanne, 1015 Switzerland.

[6] Center for Studies of Air Quality and Climate Change, Institute of Chemical Engineering Sciences, Foundation for Research and Technology Hellas, Patras,26504 Greece.

[a] Now at: U.S. Army DEVCOM CBC, Aberdeen Proving Ground, Maryland, 21010 USA

[b] Now at: Sustainable Energy and Environment Thrust, The Hong Kong University of Science and Technology (Guangzhou), Guangzhou, 511400 China

Table S1. Detailed description of the indoor experiments.

Date	Start time	Stop time	period	Emission type
2022-01-25	2:15:00 PM	5:15:00 PM	Active Emissions	Pellet Stove
2022-01-25	5:15:00 PM	8:15:00 PM	ConcDecay	Pellet Stove
2022-01-28	4:12:00 PM	7:20:00 PM	Active Emissions	Pellet Stove
2022-01-28	7:20:00 PM	9:20:00 PM	ConcDecay	Pellet Stove
2022-01-29	12:03:00 PM	1:00:00 PM	Active Emissions	Cooking[1]
2022-01-29	1:00:00 PM	2:00:00 PM	ConcDecay	Cooking
2022-01-29	3:00:00 PM	3:14:00 PM	Active Emissions	Cooking[2]
2022-01-29	3:14:00 PM	6:14:00 PM	ConcDecay	Cooking
2022-01-30	1:15:00 PM	1:35:00 PM	Active Emissions	Cooking[3]
2022-01-30	1:35:00 PM	3:00:00 PM	ConcDecay	Cooking
2022-01-30	3:00:00 PM	4:15:00 PM	Active Emissions	Cooking[3]
2022-01-30	4:15:00 PM	6:15:00 PM	ConcDecay	Cooking
2022-01-31	3:15:00 PM	6:15:00 PM	Active Emissions	Pellet Stove
2022-01-31	6:15:00 PM	9:15:00 PM	ConcDecay	Pellet Stove
2022-02-03	2:55:00 PM	5:20:00 PM	Active Emissions	Pellet Stove
2022-02-03	5:20:00 PM	8:20:00 PM	ConcDecay	Pellet Stove
2022-02-05	12:12:00 PM	3:12:00 PM	Active Emissions	Pellet Stove
2022-02-05	3:12:00 PM	6:12:00 PM	ConcDecay	Pellet Stove
2022-02-07	10:15:00 AM	1:15:00 PM	Active Emissions	Mixed
2022-02-07	1:15:00 PM	6:30:00 PM	ConcDecay	Mixed
2022-02-08	9:15:00 AM	12:01:00 PM	Active Emissions	Mixed
2022-02-08	12:01:00 PM	4:01:00 PM	ConcDecay	Mixed
2022-02-09	10:15:00 AM	1:01:00 PM	Active Emissions	Mixed
2022-02-09	1:01:00 PM	5:01:00 PM	ConcDecay	Mixed
2022-02-10	11:30:00 AM	2:14:00 PM	Active Emissions	Mixed
2022-02-10	2:14:00 PM	6:14:00 PM	ConcDecay	Mixed
2022-02-11	10:30:00 AM	1:38:00 PM	Active Emissions	Pellet Stove
2022-02-11	1:38:00 PM	5:38:00 PM	ConcDecay	Pellet Stove
2022-02-16	3:00:00 PM	6:07:00 PM	Active Emissions	Mixed
2022-02-16	6:07:00 PM	10:07:00 PM	ConcDecay	Mixed
2022-02-18	1:00:00 PM	3:09:00 PM	Active Emissions	Mixed
2022-02-18	3:09:00 PM	6:30:00 PM	ConcDecay	Mixed
2022-02-18	8:30:00 PM	8:50:00 PM	Active Emissions	Incense
2022-02-18	8:50:00 PM	11:50:00 PM	ConcDecay	Incense
2022-02-21	1:30:00 PM	4:40:00 PM	Active Emissions	Pellet Stove
2022-02-21	4:40:00 PM	7:45:00 PM	ConcDecay	Pellet Stove
2022-02-21	7:45:00 PM	8:30:00 PM	Active Emissions	Incense

2022-02-21	8:30:00 PM	11:30:00 PM	ConcDecay	Incense
2022-02-22	10:00:00 AM	1:12:00 PM	Active Emissions	Pellet Stove
2022-02-22	1:12:00 PM	4:12:00 PM	ConcDecay	Pellet Stove
2022-02-23	11:30:00 AM	12:20:00 PM	Active Emissions	Incense
2022-02-23	12:20:00 PM	1:30:00 PM	ConcDecay	Incense
2022-02-23	1:30:00 PM	4:44:00 PM	Active Emissions	Pellet Stove
2022-02-23	4:44:00 PM	7:45:00 PM	ConcDecay	Pellet Stove
2022-02-23	8:00:00 PM	8:30:00 PM	Active Emissions	Car (Outdoor Source)
2022-02-24	10:30:00 AM	11:20:00 AM	Active Emissions	Incense
2022-02-24	11:20:00 AM	2:20:00 PM	ConcDecay	Incense

[1] Pasta boil and sauce simmer.

[2] Oil aerosol generation: add ¼ cup of vegetable oil to the sauté pan and add water to the hot oil.

[3] Both Pasta boil and oil aerosol generation.

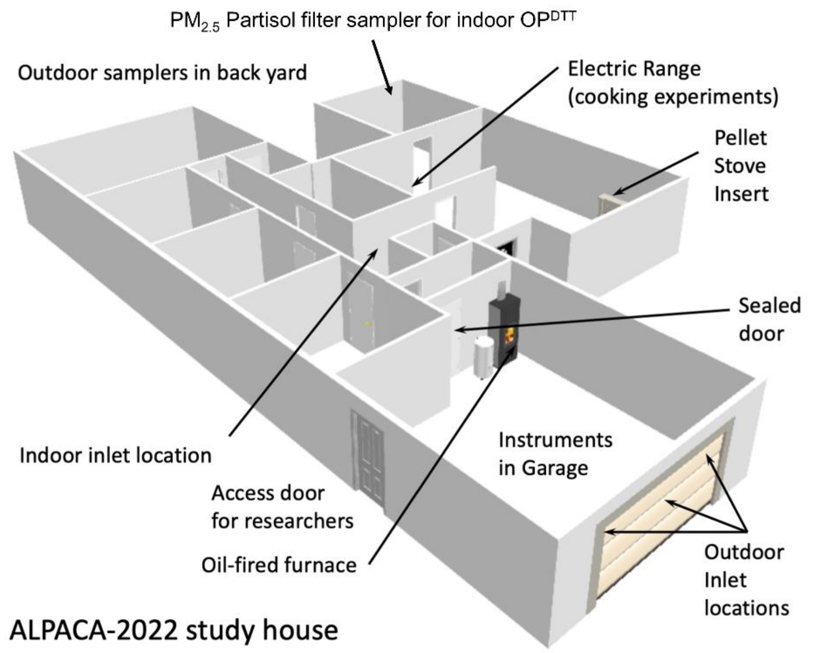

Figure S1. A 3-D rendering of the one-story house's plan showing locations of inlets and key indoor sources and inlet locations. The house was in a suburban area of Fairbanks, Alaska.

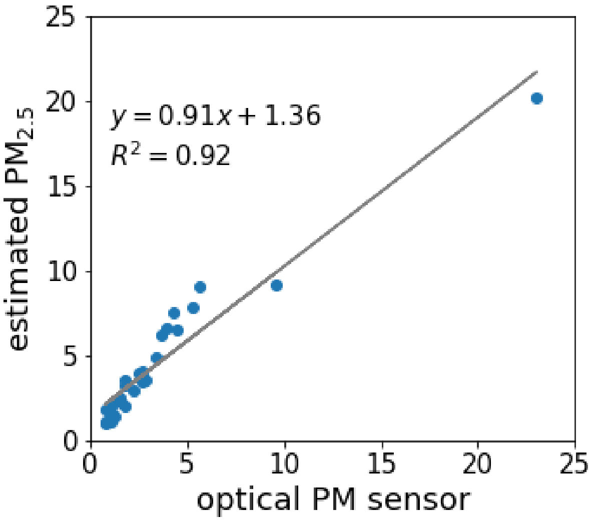

Figure S2. Comparison between indoor $PM_{2.5}$ mass concentration estimated by PM_1+BC and $PM_{2.5}$ measurement using a low-cost optical PM sensor merged to the filter sampling time, Orthogonal regression was applied.

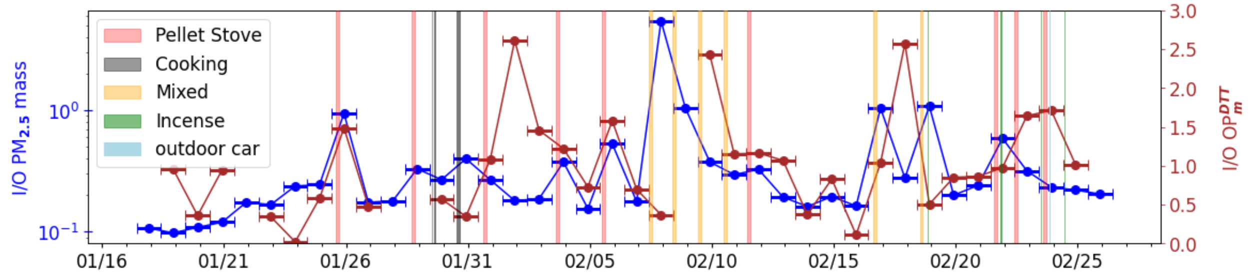

Figure S3. Time series of indoor-outdoor (I/O) $PM_{2.5}$ mass, I/O OP_m^{DTT} and the time slot of different experiments.

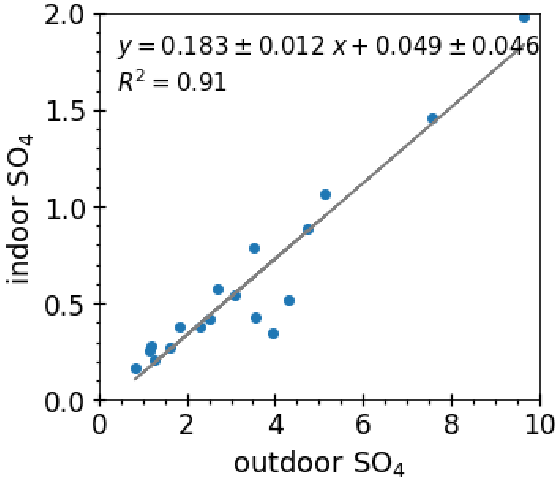

Figure S4. Daily average PM_1 sulfate concentrations measured indoors and outdoors with an Aerosol Mass Spectrometer (AMS) merged to the filter sampling time, during periods of no indoor activities (Background and No-experiments). Orthogonal regression was applied. Background refers to the period at the start of the study prior to any indoor perturbation experiments. No experiments are days during the study when there was no perturbation experiment, but experiments had occurred on previous days.
